# Identification of Rice m^6^A Methyltransferase Complex Subunits MTA/MTB and Their Effects on Heading Date and Grain Weight

**DOI:** 10.3390/plants15172667

**Published:** 2026-08-31

**Authors:** Chengxuan Li, Mengjun Zhang, Duanmu Zhao, Xue Cao, Hengzhi Wang, Mojia Liu, Tong Zhang, Wenyi Wang, Jian Wu

**Affiliations:** 1 Guangdong Laboratory for Lingnan Modern Agriculture, Guangdong Basic Research Center of Excellence for Precise Breeding of Future Crops, Institute of Water-Saving and Drought-Resistance Rice Green Industry, College of Agriculture, South China Agricultural University, Guangzhou 510642, China; muyan20210601@163.com (C.L.); mjluckyforever@163.com (M.Z.); 17622735990@163.com (D.Z.); gdxsz1225@163.com (H.W.); mikemorton0926@163.com (M.L.); 2 Guangdong Province Key Laboratory of Microbial Signals and Disease Control, College of Plant Protection, South China Agricultural University, Guangzhou 510642, China; caoxue@scau.edu.cn (X.C.); zhangtong@scau.edu.cn (T.Z.)

**Keywords:** N^6^-methyladenosine, rice, heading date, m^6^A writer complex

## Abstract

N^6^-methyladenosine (m^6^A) is a vital epitranscriptomic modification that regulates plant development and stress responses. In rice (*Oryza sativa* L.), the identity of the catalytic subunit (MTA) of the core m^6^A writer complex has remained controversial because of inconsistent annotations based primarily on sequence homology. Here, phylogenetic analysis identified LOC_Os02g45110 as the putative catalytic subunit OsMTA, whereas LOC_Os01g16180 and LOC_Os03g05420 were classified as the structural subunits OsMTB1 and OsMTB2, respectively. All three proteins showed nuclear enrichment. Yeast two-hybrid, luciferase complementation imaging, and bimolecular fluorescence complementation assays demonstrated that OsMTA interacts independently with OsMTB1 and OsMTB2. Moreover, transient co-expression of *OsMTA* with either *OsMTB1* or *OsMTB2* increased global m^6^A levels in a heterologous system. No homozygous plants carrying frameshift mutations in *OsMTA* were obtained, suggesting that complete loss of *OsMTA* function is lethal. Knockdown of *OsMTA* or CRISPR/Cas9-mediated knockout of *OsMTB1* and *OsMTB2* significantly delayed heading date and reduced 1000-grain weight. Conversely, overexpressing *OsMTA* accelerated heading, whereas overexpressing *OsMTB1* or *OsMTB2* generally accelerated heading but significantly reduced 1000-grain weight. Together, these findings define the core composition of the rice m^6^A writer complex and reveal its effects on heading date and grain weight.

## 1. Introduction

N^6^-methyladenosine (m^6^A) was first detected in poly(A) RNA fractions in the 1970s [1]. It has since been established as the most prevalent internal modification in eukaryotic mRNAs [2], playing essential roles in RNA processing, transport, translation, and degradation, as well as in plant growth, development, and stress responses [3]. The dynamic deposition, interpretation, and removal of m^6^A are precisely orchestrated by three classes of proteins: writers (methyltransferases), readers (RNA-binding proteins), and erasers (demethylases). In the canonical m^6^A writer complex, the catalytic core is composed of a catalytic subunit (MTA) and a structural subunit (MTB) [4,5].

In rice (*Oryza sativa* L.), the m^6^A regulatory landscape has been extensively characterized. Single-base resolution profiling has mapped 205,691 m^6^A sites across 22,574 rice genes, mainly enriched near stop codons and within 3′ untranslated regions (UTRs) [6]. The broader rice m^6^A regulatory network comprises at least 124 components, including 7 writers, 22 readers, and 95 erasers [7]. Functionally, m^6^A is indispensable for rice reproductive development. OsFIP37 and the protein encoded by *LOC_Os02g45110* form an m^6^A writer complex essential for male gametogenesis; its loss causes microspore degeneration and abnormal meiosis [8]. Additional m^6^A components also contribute to male fertility through regulating tapetal programmed cell death (PCD) and pollen development [9,10]. Furthermore, m^6^A readers regulate flowering through distinct mechanisms, such as stabilizing or sequestering flowering-related transcripts [11,12]. Beyond transcription, m^6^A also influences translation: OseIF3h interacts with the *LOC_Os02g45110*-encoded protein to regulate photosynthesis and energy metabolism genes, linking m^6^A to translation initiation [13]. The agricultural potential of m^6^A manipulation has already been demonstrated: heterologous expression of the human m^6^A demethylase FTO in rice reduces m^6^A levels and increases yield by approximately 50% in field trials [14].

In addition to development, m^6^A function is a hallmark of rice stress adaptation. Under salt stress, LOC_Os01g16180-mediated methylation regulates seed germination [15]. Drought stress induces the expression of OsALKBH eraser genes [16], while chromium stress leads to hypomethylation of *OsHMT9.1*, the knockout of which enhances Cr tolerance via trace-element homeostasis [17]. Temperature extremes also remodel the m^6^A landscape: cold stress alters the m^6^A/A ratio and the expression of m^6^A writers and readers [18,19], while heat stress upregulates the m^6^A readers OsECT3 and OsECT6 [20]. Biotic stress triggers m^6^A reprogramming on stress-related transcripts during viral infection and insect herbivory [21,22].

Despite these extensive insights, a fundamental controversy remains in the field: the true catalytic subunit (MTA) of the rice m^6^A writer complex has not been conclusively identified. The same protein encoded by *LOC_Os02g45110* has been variably annotated as OsMTA [18], OsMTA2 [8,13], OsMTA-3 [7], or OsMTB [23] across different studies. This confusion arose because previous studies relied primarily on sequence similarity, without considering functional divergence between the catalytic MTA and the structural MTB subunits. By contrast, in *Arabidopsis thaliana*, the core writer complex is well-defined, consisting of MTA, MTB, and FIP37, with disruption of MTA causing embryonic lethality, and loss of MTB or FIP37 also leading to severe developmental defects [4,24,25]. This disparity raises a critical question: which rice proteins are true orthologs of MTA and MTB?

In this study, we identified OsMTA, OsMTB1, and OsMTB2 as core components of the rice m^6^A writer complex through phylogenetic, biochemical, and genetic analyses. All three proteins showed nuclear enrichment, and the putative catalytic subunit OsMTA interacts with either structural subunit OsMTB1 or OsMTB2 to form a functional complex that increases global m^6^A levels in the heterologous system. Genetic analyses suggested that complete loss of *OsMTA* function is lethal, and that *OsMTA*, *OsMTB1* and *OsMTB2* are associated with heading date and grain weight. These findings clarify the identities of the rice MTA and MTB proteins and provide a framework for understanding m^6^A writer complex assembly and its effects on heading date and grain weight.

## 2. Results

### 2.1. Phylogenetic, Conserved Domain, and Selection Analysis of m^6^A Methyltransferases MTA and MTB Across Different Species

**Figure 1:**
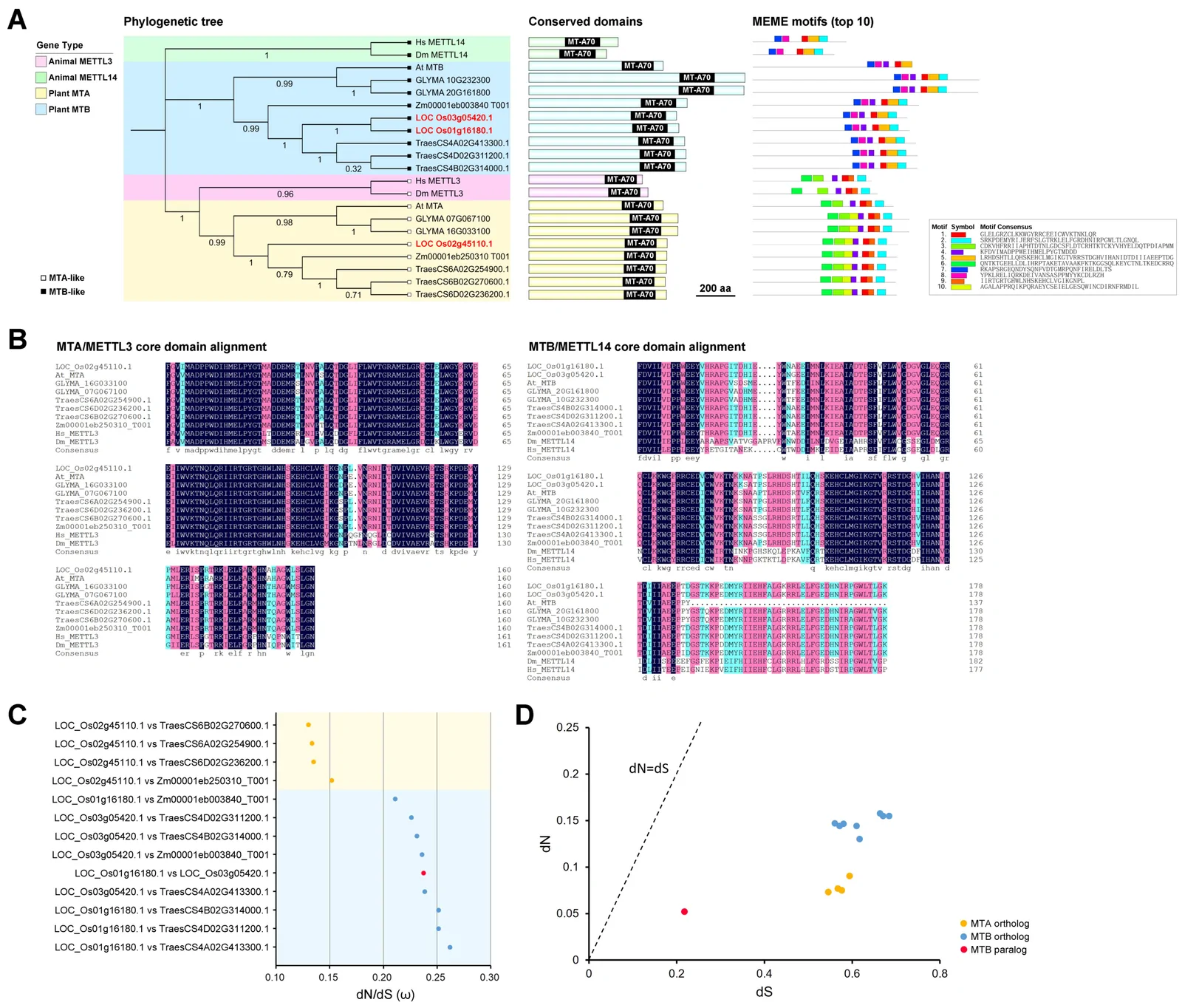
Phylogenetic, structural, and selection analysis of MTA and MTB proteins from *O. sativa*, *A. thaliana*, *T. aestivum*, *Z. mays*, *G. max*, *D. melanogaster* and *H. sapiens*. (**A**) Phylogenetic tree of MTA and MTB homologs from seven species, with conserved MT-A70 domain organization and MEME motif composition shown for these proteins. LOC_Os02g45110, LOC_Os01g16180, LOC_Os03g05420 are highlighted in red and bold. (**B**) Multiple sequence alignment of MT-A70 core domains. (**C**) Horizontal dot plot of dN/dS ratios for rice MTA and MTB genes against grass orthologs (maize, wheat), as well as the rice MTB paralog pair, grouped by the MTA and MTB families. (**D**) dN versus dS scatter plot based on the data shown in Figure 1C. The dotted line represents dN = dS; all points fall below this line, indicating purifying selection.

To determine the MTA and MTB subunits in rice, we performed a phylogenetic analysis of MTA and MTB homologs across multiple model plants and animals. Protein sequences from rice (*Oryza sativa* L.), *Arabidopsis* (*Arabidopsis thaliana*), wheat (*Triticum aestivum*), maize (*Zea mays*), soybean (*Glycine max*), fruit fly (*Drosophila melanogaster*), and human (*Homo sapiens*) were aligned to construct a phylogenetic tree (Figure 1A). The phylogenetic tree showed that MTA and MTB family members exhibit clear branch clustering, forming four main evolutionary clades based on sequence conservation and evolutionary relationships. For rice, LOC_Os02g45110 clustered within the plant MTA clade represented by *Arabidopsis* At_MTA, indicating high evolutionary conservation; LOC_Os01g16180 and LOC_Os03g05420 clustered within the plant MTB clade, with the two rice proteins forming a well-supported sister pair, suggesting possible functional divergence. Many nodes had high bootstrap support values (>0.9), supporting the inferred relationships. Our clustering results for these three m^6^A writer proteins are consistent with a recent study [18]. Domain analysis confirmed that all three rice proteins retain the conserved MT-A70 methyltransferase domain (PF05063): OsMTA (706 aa) contains the domain at residues 491–651, while OsMTB1 (766 aa) and OsMTB2 (754 aa) contain it at positions 529–707 and 521–699, respectively (Figure 1A). MEME motif analysis revealed family-specific conserved sequence blocks, with OsMTB1 and OsMTB2 showing similar motif composition consistent with their close phylogenetic relationship (Figure 1A). Multiple sequence alignment of the extracted MT-A70 core domains further showed that OsMTA conserves the catalytic DPPW motif, whereas OsMTB1 and OsMTB2 display conservation patterns characteristic of MTB family members (Figure 1B), which are generally considered to function as structural components of the m^6^A writer complex. These results are consistent with the designation of LOC_Os02g45110 as the putative catalytic subunit OsMTA, and LOC_Os01g16180 and LOC_Os03g05420 as the structural subunits OsMTB1 and OsMTB2, respectively.

To investigate the evolutionary constraints acting on plant MTA and MTB homologs, we performed pairwise dN/dS analysis of rice MTA and MTB genes against their orthologs from other grass species (maize and wheat) (Figure 1C,D). Cross-species comparisons between monocots and dicots were attempted but yielded dS estimates exceeding the saturation threshold (dS > 2), and were therefore excluded from further analysis and visualization; these conclusions therefore apply primarily to within-Poaceae comparisons. Analysis based on the dN/dS ratios showed that all retained MTA and MTB ortholog pairs exhibit values well below 1, indicating that these genes have evolved under strong purifying selection. For the MTA family, dN/dS values between rice and grass species ranged from 0.130 to 0.152 (mean 0.138), suggesting strong functional constraint across Poaceae. For the MTB family, rice-grass ortholog comparisons yielded dN/dS values ranging from 0.210 to 0.262 (mean 0.238), also consistent with purifying selection. The OsMTB1–OsMTB2 paralog pair showed a dN/dS ratio of 0.2374 (dN = 0.0517, dS = 0.2176), indicating that the duplicated rice MTB genes have been subject to purifying selection since their divergence. No evidence of positive selection (dN/dS > 1) was detected in any comparison. These results are consistent with purifying selection and conserved functions of MTA and MTB genes in grasses.

### 2.2. OsMTA and OsMTB1/2 Show Nuclear Enrichment and Relatively High Transcript Levels in Stems and Seeds

**Figure 2:**
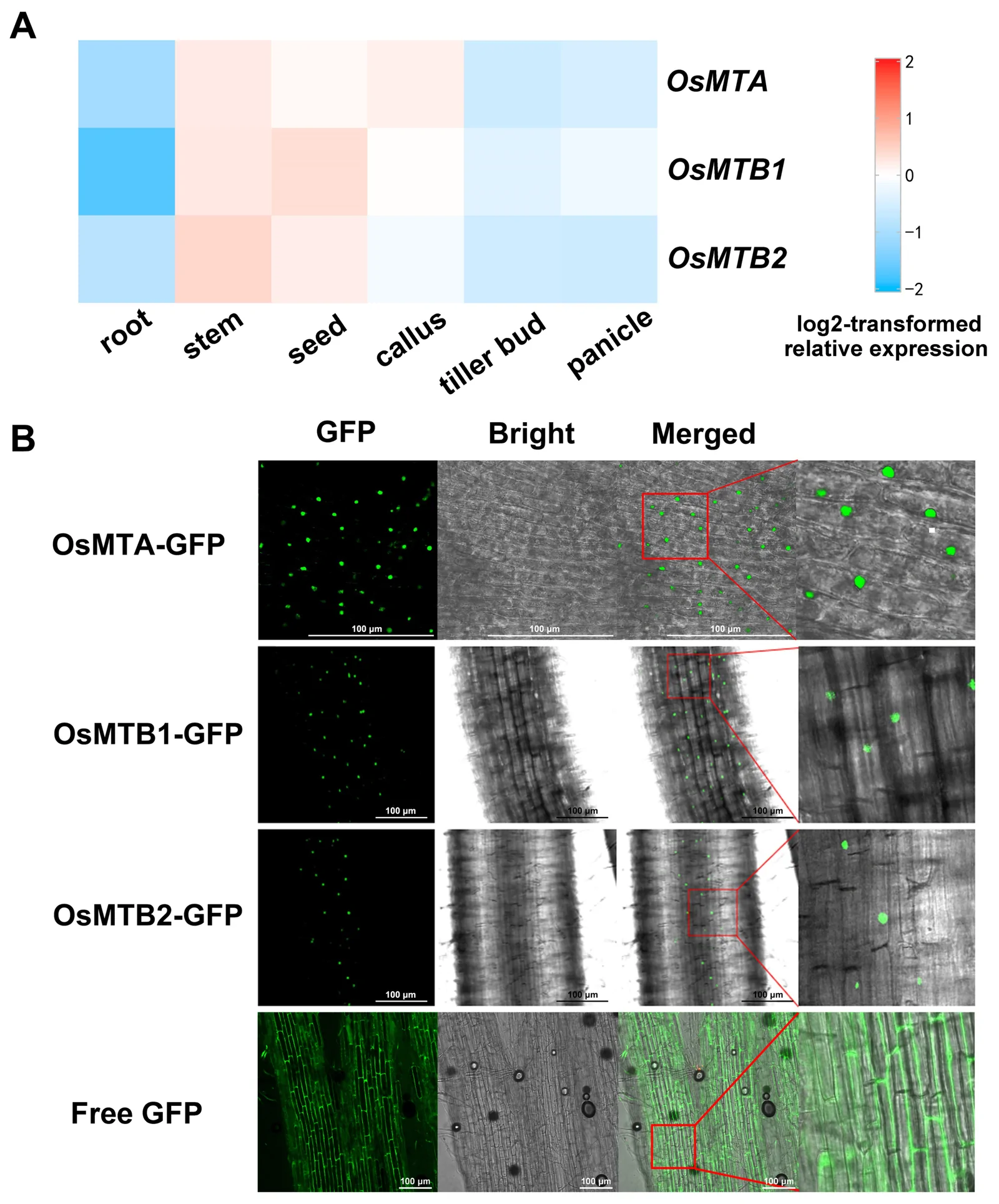
Expression patterns and subcellular localization of OsMTA, OsMTB1, and OsMTB2. (**A**) Heatmap showing expression levels of *OsMTA*, *OsMTB1*, and *OsMTB2* in various tissues. The color key represents log_2_-transformed relative expression values. (**B**) Confocal images of rice root tip cells expressing OsMTA-GFP, OsMTB1-GFP, and OsMTB2-GFP fusion proteins. Free GFP was used as a control. Scale bars, 100 μm.

Heatmap analysis (Figure 2A) revealed that *OsMTA*, *OsMTB1* and *OsMTB2* showed relatively high transcript levels in stems and seeds, raising the possibility that the m^6^A writer complex may contribute to source-sink-related processes, although this remains to be tested experimentally. Given their highly similar expression profiles, these three components may function as subunits of larger heteromeric m^6^A writer complexes.

To investigate the subcellular localization of OsMTA, OsMTB1, and OsMTB2, we generated transgenic plants overexpressing OsMTA-GFP, OsMTB1-GFP and OsMTB2-GFP fusion proteins driven by the *CaMV35S* promoter. Transgenic plants expressing free GFP were used as a control for the localization pattern. Observation of root tip cells under a confocal laser scanning microscope revealed that all three proteins showed nuclear enrichment (Figure 2B), whereas free GFP was distributed throughout both the nucleus and cytoplasm. This nuclear enrichment is consistent with the role of these proteins in post-transcriptional m^6^A modification and further supports their function as components of the m^6^A writer complex.

### 2.3. OsMTA Interacts with OsMTB1/OsMTB2 to Enhance m^6^A Levels

**Figure 3:**
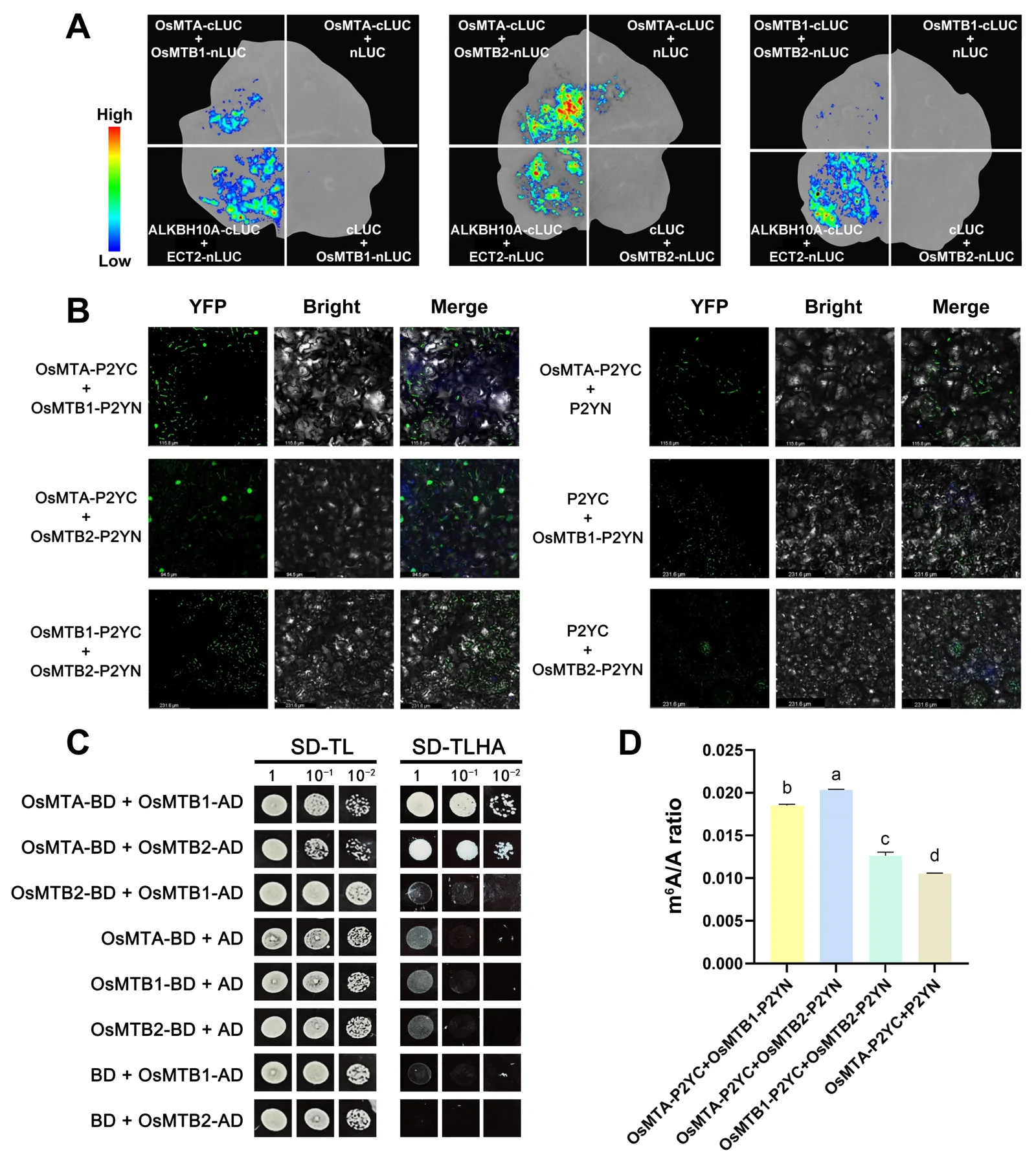
Interaction assays of OsMTA, OsMTB1, OsMTB2, and m^6^A level determination. (**A**) LCI assay verifying the interaction of OsMTA and OsMTBs in *N. benthamiana* leaves. The known interacting *Arabidopsis* proteins ALKBH10A-cLUC + ECT2-nLUC were used as a positive control [26], while nLUC and cLUC were used as a negative control. nLUC, N terminus of LUC; cLUC, C terminus of LUC. The color scale bar represents luminescence intensity from low to high. (**B**) BiFC assay revealing the interaction between OsMTA and OsMTBs in *N. benthamiana* leaves. Scale bars are indicated individually for each panel. (**C**) OsMTA interacts with OsMTB1 and OsMTB2 in yeast. Transformed yeast cells were grown on SD-Leu/Trp and SD-Leu/-Trp/-His/-Ade medium, respectively. (**D**) m^6^A/A ratio in agro-infiltrated *N. benthamiana* leaves. Data are presented as mean ± SD. Different lowercase letters indicate statistically significant differences among treatments (LSD test, *p* < 0.05).

In animals and *Arabidopsis*, MTA and MTB have been shown to physically interact with each other [4,5]. To investigate whether the rice homologs OsMTA, OsMTB1, and OsMTB2 exhibit similar interactions, we conducted a yeast two-hybrid (Y2H) assay. The results showed that OsMTA physically interacts with OsMTB1 and OsMTB2 in yeast, whereas no interaction was detected between OsMTB1 and OsMTB2 (Figure 3C). No autoactivation was observed in the empty bait/prey combinations. This finding suggests that OsMTB1 and OsMTB2 cannot simultaneously function as both the catalytic and structural subunits of the writer complex, further supporting their roles as structural MTB proteins rather than the catalytic MTA. Subsequently, luciferase complementation imaging (LCI) and bimolecular fluorescence complementation (BiFC) in *Nicotiana benthamiana* confirmed these interactions *in planta* (Figure 3A,B).

We also used the same BiFC transient expression system in *Nicotiana benthamiana* to assess the effects of OsMTA and OsMTB1/2 on m^6^A modification. Co-expression of *OsMTA* with either *OsMTB1* or *OsMTB2* resulted in a significantly higher m^6^A/A ratio than expression of *OsMTA* alone or co-expression of *OsMTB1* and *OsMTB2* (Figure 3D). The m^6^A/A ratios in the OsMTA-P2YC + OsMTB1-P2YN and OsMTA-P2YC + OsMTB2-P2YN treatments were approximately 1.75 and 1.93 times that of OsMTA-P2YC + P2YN treatment, respectively. These results indicate that OsMTA, together with OsMTB1 or OsMTB2, forms a functional complex that increases global m^6^A levels in the heterologous system. Together, these data indicate that OsMTA interacts with OsMTB1 and OsMTB2 within the nucleus of living plant cells, forming the core structure of the plant m^6^A writer complex and contributing to m^6^A modification. Collectively, our interaction and functional assays support LOC_Os02g45110 as the putative catalytic MTA subunit, and LOC_Os01g16180/LOC_Os03g05420 as the structural MTB subunits in rice.

### 2.4. OsMTA, OsMTB1 and OsMTB2 Affect Heading Date and 1000-Grain Weight

**Figure 4:**
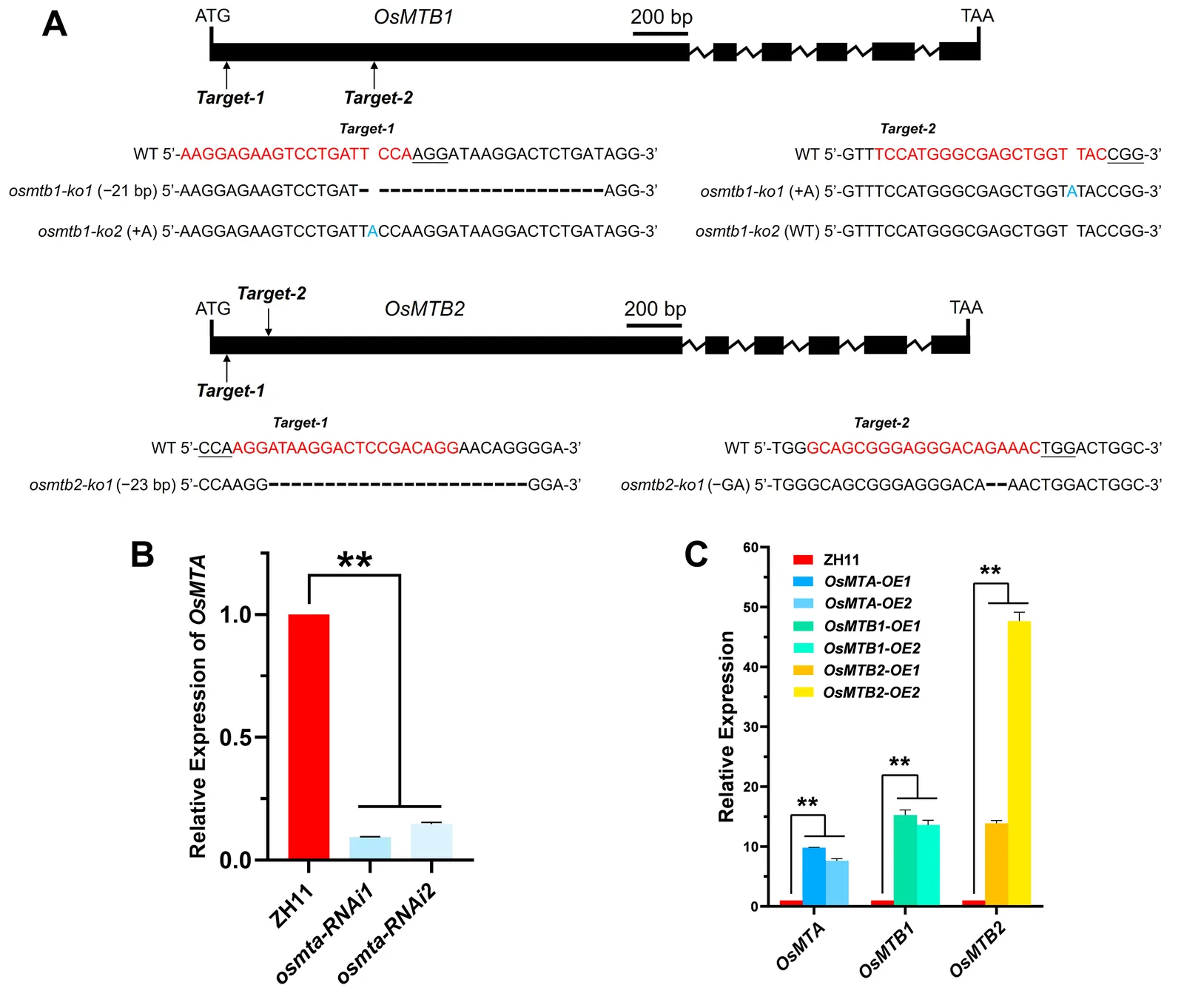
Genetic information of *OsMTA*, *OsMTB1* and *OsMTB2* transgenic lines. (**A**) Sequence alignment of knockout lines mutant at *OsMTB1* and *OsMTB2* loci. The sequencing results revealed a 21-bp deletion accompanied by an adenine insertion in *osmtb1-ko1*, an adenine insertion in *osmtb1-ko2*, and a 23-bp deletion accompanied by a GA deletion in *osmtb2-ko1*. The target sites are highlighted in red. (**B**) Relative expression of *OsMTA* in ZH11 and RNAi lines. (**C**) Relative expression of *OsMTA*, *OsMTB1* and *OsMTB2* in ZH11 and overexpression lines. Data are presented as mean ± SD. Significant differences were determined by Student’s *t*-test, **p < 0.01.

To investigate the roles of *OsMTA* and *OsMTB* in rice development, we generated loss-of-function and gain-of-function lines in the ZH11 background. Knockout lines were created using the CRISPR/Cas9 approach. For each gene, two single-guide RNAs were designed targeting different regions of the early coding sequence to create mutants. The resulting loss-of-function mutants of *OsMTB1* and *OsMTB2* without the CRISPR-Cas9 construct were named *osmtb1-ko1*, *osmtb1-ko2* and *osmtb2-ko1* (Figure 4A). An additional independent *OsMTB2* knockout line, *osmtb2-ko2*, was also obtained and characterized in a separate growth season (Figure S1). We generated five independent *OsMTA*-edited lines (Figure S1). However, despite screening up to the T2 generation, no homozygous frameshift knockout lines were obtained. Therefore, we generated silencing lines named *osmta-RNAi1* and *osmta-RNAi2*. Overexpression lines for all three genes were generated using the maize ubiquitin promoter. qRT-PCR analysis confirmed that the target genes were substantially upregulated in the overexpression lines and significantly downregulated in the RNAi lines (Figure 4B,C). We also observed GFP fluorescence in the seedling-stage plants of *OsMTB1-OE1* and *OsMTB1-OE2* (Figure S4).

**Figure 5:**
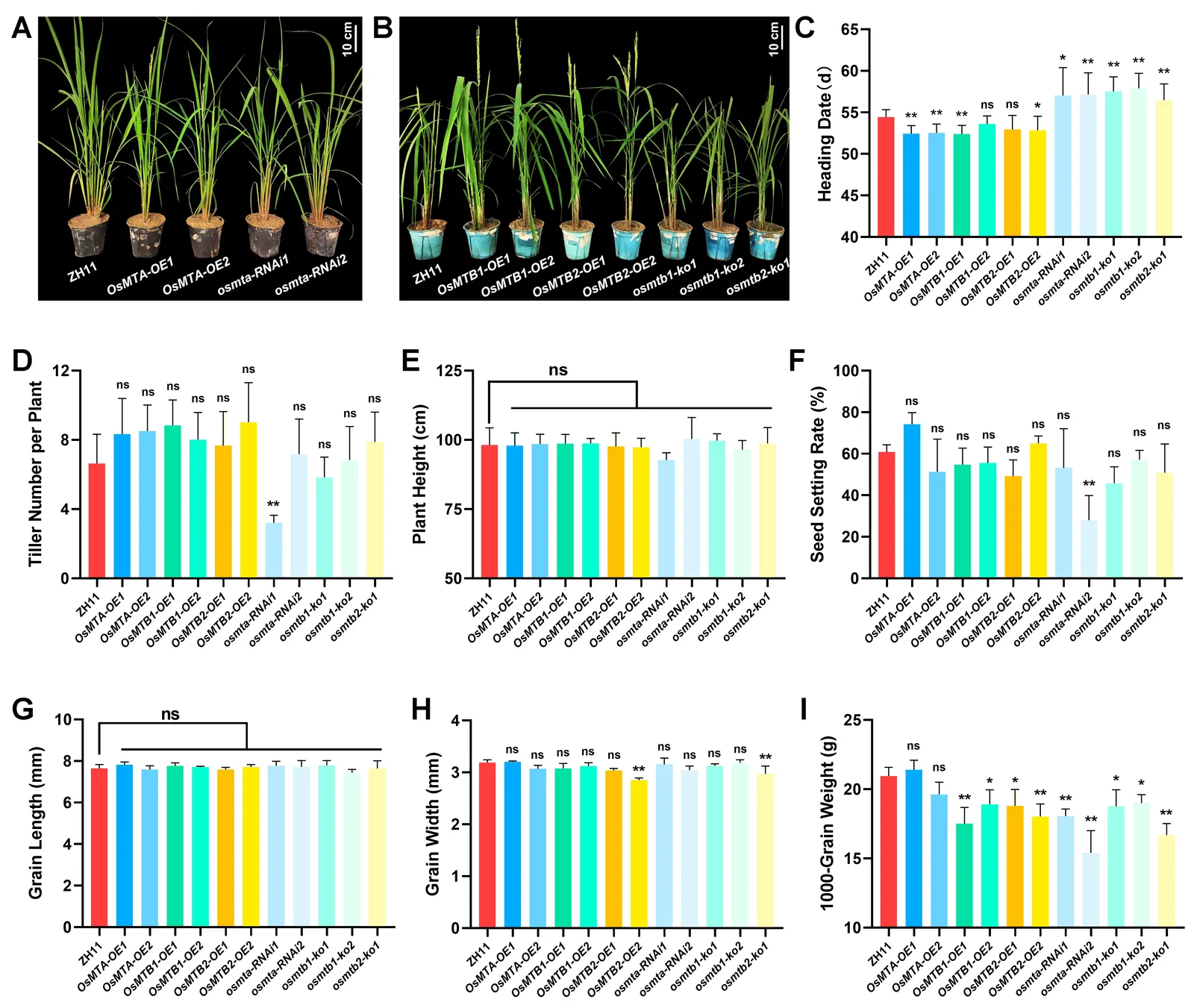
Agronomic traits of transgenic lines. All agronomic measurements were obtained from field-grown plants. (**A**–**C**) Heading dates of transgenic lines compared with ZH11. Overexpression lines generally exhibited an earlier heading date, while loss-of-function lines showed a later heading date. (**D**–**H**) Tiller number per plant (**D**), plant height (**E**), seed-setting rate (**F**), grain length (**G**) and grain width (**H**) of transgenic lines compared with ZH11. (**I**) 1000-grain weight (TGW) of transgenic lines compared with ZH11. All genetic lines except *OsMTA-OE1* and *OsMTA-OE2* showed significantly reduced TGW. Data are presented as mean ± SD. Significant differences were determined by one-way ANOVA followed by Dunnett’s test, * *p* < 0.05, ** *p* < 0.01, ns = no significance.

Phenotypic analysis revealed that both loss- and gain-of-function of these genes primarily affected reproductive development. Knockdown of *OsMTA* and knockout of *OsMTB1* or *OsMTB2* consistently delayed heading. Conversely, overexpression of *OsMTA*, *OsMTB1*, and *OsMTB2* generally accelerated heading (Figure 5A–C and Figure S5A). Although *OsMTB1-OE2* and *OsMTB2-OE1* did not reach the significance threshold, their trends were consistent. This effect was further validated in a hybrid background, where *OsMTB1-OE1*×HHZ exhibited a 2.5-day earlier heading date compared to the ZH11×HHZ control (Table S1 and Figure S6). Tiller number was largely unaffected except for a sharp reduction in *osmta-RNAi1* (−3.44 tillers) (Figure 5D), and plant height remained unchanged across all lines (Figure 5E and Figure S5C).

Overexpression of these genes had no adverse effect on seed-setting rate. Significant decreases in seed-setting rate were observed only in *osmta-RNAi1* and *osmtb2-ko2*, while the remaining loss-of-function lines were unaffected (Figure 5F and Figure S5D). Analysis of grain size revealed that grain length remained unchanged (Figure 5G and Figure S5E), while grain width was reduced in *OsMTB2-OE2* and *osmtb2-ko1* (Figure 5H). However, all genetic lines except *OsMTA-OE1* and *OsMTA-OE2* showed significantly reduced 1000-grain weight (Figure 5I and Figure S5G). Together, these results suggest that *OsMTA* and *OsMTB1/2* promote heading and are associated with 1000-grain weight, contributing to normal reproductive development in rice.

## 3. Discussion

The m^6^A modification is an abundant epitranscriptomic mark across eukaryotes [27]. Organisms regulate cellular m^6^A homeostasis through the coordinated actions of “writer” and “eraser” proteins, while “reader” proteins interpret this modification to modulate downstream RNA metabolism, plant development, and stress adaptation [3]. In this study, we demonstrated that OsMTA, OsMTB1, and OsMTB2 constitute the core components of the rice m^6^A writer complex. Phylogenetically, OsMTA and the two OsMTB variants cluster tightly with their corresponding orthologs in *Arabidopsis*, wheat and maize. This evolutionary grouping underscores the structural conservation of the core m^6^A writer complex across diverse plant species, consistent with the paradigm established by mammalian METTL3 and METTL14 [5].

Our biochemical and cell biology assays reinforce this evolutionary conservation. Both OsMTA and OsMTB1/2 proteins show nuclear enrichment, aligning with the established spatial framework where co-transcriptional m^6^A deposition occurs [28]. Expression profiling revealed relatively high transcript levels of *OsMTA*, *OsMTB1*, and *OsMTB2* in stems and seeds. This expression pattern raises the possibility that the m^6^A writer complex may contribute to source-sink-related processes, although this remains to be tested experimentally. Mirroring the mammalian writer complex architecture [5], we verified that OsMTA physically interacts with both OsMTB1 and OsMTB2 in yeast and *in vivo*. In transient expression assays in *Nicotiana benthamiana*, co-expression of *OsMTA* with either *OsMTB1* or *OsMTB2* resulted in significantly higher m^6^A/A ratios than expression of *OsMTA* alone or co-expression of *OsMTB1* and *OsMTB2*. This finding suggests that OsMTB1 and OsMTB2 are not merely passive accessory proteins, but are instead structural scaffolding components required for full methyltransferase activity in the heterologous system.

The failure to obtain homozygous frameshift *OsMTA* knockout lines suggests that complete loss of *OsMTA* function is lethal. A similar requirement has been documented in *Arabidopsis*, where the knockout of the MTA ortholog arrests embryonic development at the globular stage [4], highlighting the necessity of a fully functional writer complex for plant viability. The observation that both knockout and overexpression of *OsMTB1/2* reduce grain weight suggests that the MTA/MTB complex is dosage-sensitive, and that either depletion or excess of a subunit may disrupt normal complex function. However, alternative explanations, including pleiotropic effects and transgene insertion effects, cannot be excluded based on the current data. These observations are also consistent with the view that precise m^6^A regulation is required for normal reproductive development [8,9,10]. Further biochemical characterization of MTA/MTB complex assembly will be needed to clarify how the dosage of individual subunits differentially affects agronomic traits such as grain weight. Recent studies have shown that m^6^A readers and writers in rice regulate flowering through distinct mechanisms [11,12]. Our results extend these findings by demonstrating that the core m^6^A writer components *OsMTA*, *OsMTB1*, and *OsMTB2* also influence heading date, with loss-of-function delaying heading and overexpression generally accelerating it. However, the specific mechanism underlying this effect remains to be investigated.

**Figure 6:**
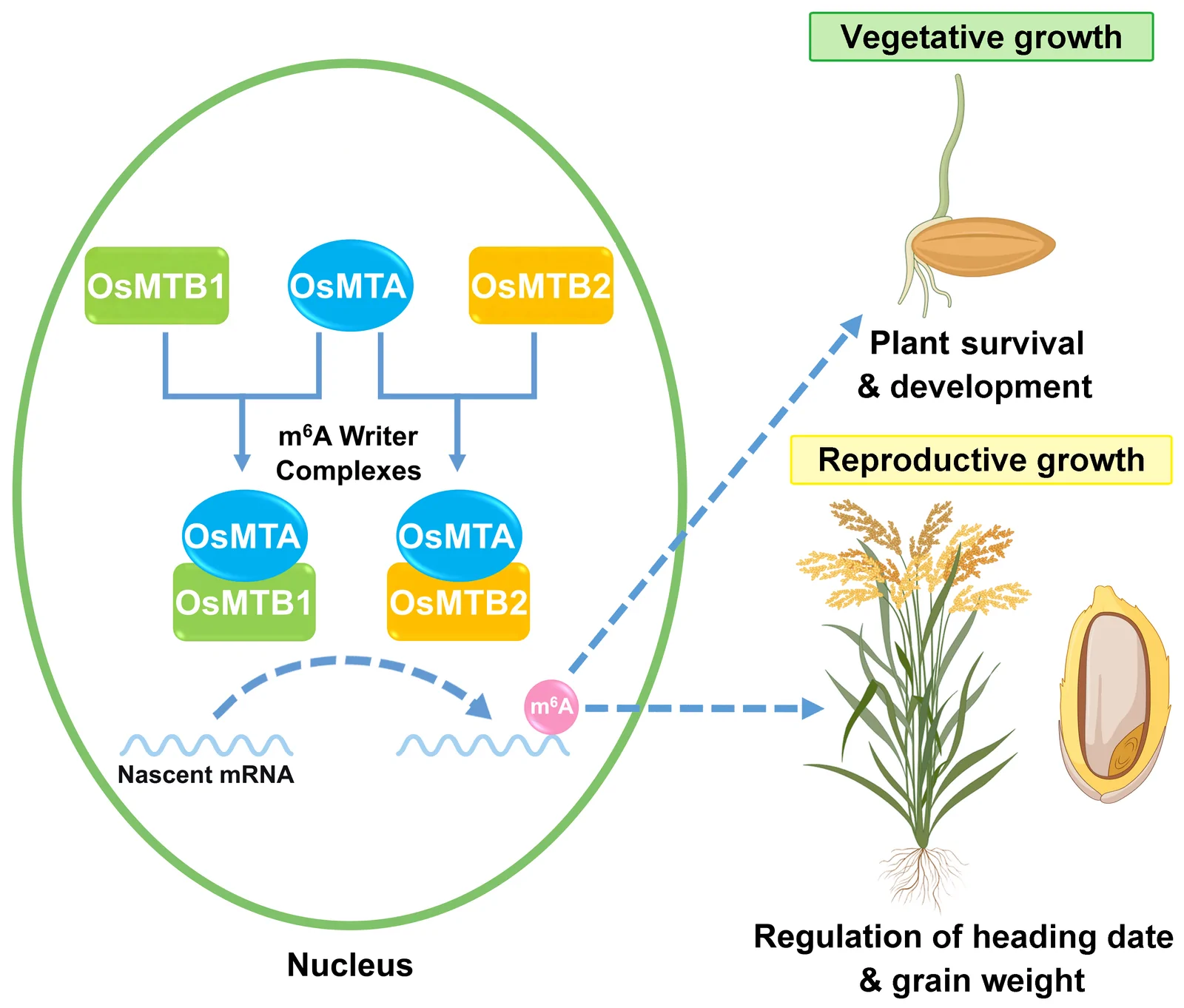
Working model of the rice m^6^A writer complex. Solid arrows indicate components supported by experimental data in this study. Dashed arrows indicate hypothesized steps that remain to be tested.

Based on our genetic and biochemical evidence, we propose a working model for the rice m^6^A writer complex (Figure 6). Within the nucleus, the putative catalytic OsMTA subunit assembles with either OsMTB1 or OsMTB2 to form a functional m^6^A writer complex. We hypothesize that this complex targets specific consensus motifs on nascent transcripts to catalyze m^6^A deposition. Our genetic data suggest that during vegetative growth, complete loss of *OsMTA* function is lethal, and that during reproductive growth, the *OsMTA*-*OsMTB1/2* complex influences agronomic traits such as heading date and grain weight. In conclusion, our study addresses the long-standing nomenclature ambiguity surrounding the rice m^6^A writer complex. By bridging the gap between evolutionary conservation and functional genetic outcomes, these findings help clarify the identities of the rice MTA and MTB proteins and provide a framework for understanding their potential roles in agronomic trait regulation. Future research should investigate the molecular link between the m^6^A writer complex and heading date regulation. This could include transcriptome-wide m^6^A profiling in stage-specific rice tissues, expression analysis of key heading-date regulators, and the potential involvement of miRNAs, to identify critical flowering-related transcripts targeted by the m^6^A writer complex for m^6^A modification.

## 4. Materials and Methods

### 4.1. Plant Materials and Growth Conditions

The rice variety used in this study was the japonica cultivar ZH11. Field experiments were performed during the early growing season (2025, March to July) at the experimental farm of the College of Agriculture, South China Agricultural University (Guangzhou, China), which is characterized by a warm subtropical monsoon climate. The average daily temperature ranged from 18 °C to 37 °C, and the natural photoperiod was approximately 12–14 h of daylight. Standard irrigation and fertilization practices were applied throughout the growing season. The *osmtb2-ko2* line was evaluated in a separate 2026 growing season and was compared with the ZH11 control from the same season; data from different years were not pooled for statistical analysis.

Agrobacterium-mediated transformation was used to generate transgenic lines. Transformed callus was selected on a medium containing hygromycin. For knockout plants, T1 plants were confirmed by PCR using *Cas9*-specific primers (forward: 5′-CTGACGCTAACCTCGACAAG-3′; reverse: 5′-CCGATCTAGTAACATAGATGACACC-3′) to be free of the *Cas9* transgene (Figure S2), and T2 plants were used for phenotypic analysis. For RNAi and overexpression lines, T1 plants were confirmed by qRT-PCR for target gene expression, and T2 plants were used for phenotypic analysis. Transgene copy number and systematic off-target analysis were not assessed.

### 4.2. Phylogenetic, Conserved Domain, and Selection Analysis

Protein sequences of MTA and MTB homologs from *Oryza sativa*, *Arabidopsis thaliana*, *Triticum aestivum*, *Zea mays* and *Glycine max* were retrieved from the Rice Genome Annotation Project (RGAP, https://rice.uga.edu/ (accessed on 28 July 2026)) and EnsemblPlants (https://plants.ensembl.org/ (accessed on 13 July 2026)). Protein sequences from *Drosophila melanogaster* and *Homo sapiens* were obtained from the NCBI database (https://www.ncbi.nlm.nih.gov/ (accessed on 28 July 2026)).

Multiple sequence alignment was performed using MAFFT v7.490 (RIKEN, Wako, Saitama, Japan) with the L-INS-i algorithm. Poorly aligned positions were removed by filtering columns with >50% gap occupancy. A phylogenetic tree was constructed using the neighbor-joining method with 1000 bootstrap replicates in MEGA v10.2.1 (Pennsylvania State University, University Park, PA, USA). The tree was visualized using iTOL (https://itol.embl.de/ (accessed on 28 July 2026)).

Conserved domains were annotated by searching the Pfam-A database using HMMER hmmscan (domain independent E-value threshold of 1 × 10^−5^); all sequences were confirmed to contain the MT-A70 methyltransferase domain (PF05063). The domain architecture was visualized using IBS 1.0.3. Multiple sequence alignment of the MT-A70 domain regions was performed and visualized using DNAMAN v10.0. De novo motif discovery of the methyltransferase core regions was performed using MEME v5.5.7 (https://meme-suite.org/meme/ (accessed on 28 July 2026)) with the zoops model, a motif width of 6–50 amino acids, and an E-value threshold of 1 × 10^−5^.

For selection analysis, protein sequences were aligned separately for the MTA and MTB families using MAFFT L-INS-i. Codon-based alignments were generated by mapping CDS onto the protein alignments using PAL2NAL (http://www.bork.embl.de/pal2nal/ (accessed on 13 July 2026)). Pairwise nonsynonymous (dN) and synonymous (dS) substitution rates were estimated using the Nei-Gojobori method with Jukes-Cantor correction. Estimates with dS > 2 were flagged as saturated and excluded from further interpretation. The OsMTB1–OsMTB2 paralog pair was analyzed separately. Visualization was performed using Python v3.8 (Python Software Foundation, Wilmington, DE, USA) with the Bio.Phylo and matplotlib libraries, and R v4.3.3 (R Foundation for Statistical Computing, Vienna, Austria) with the ggplot2 package.

### 4.3. RNA Extraction and qRT-PCR

Total RNA was extracted from multiple tissues, including root, stem, callus, tiller bud, panicle, and seed, with TRIzol reagent (Invitrogen, Thermo Fisher Scientific, Waltham, MA, USA) according to the manufacturer’s protocol. Reverse transcription to generate first-strand cDNA was carried out using HiScript Reverse Transcriptase (Vazyme, Nanjing, China). Quantitative real-time PCR (qRT-PCR) was conducted with Fast SYBR Green Master Mix (Thermo Fisher Scientific, Waltham, MA, USA). The rice *OsActin1* gene (*LOC_Os03g50885*) served as the internal control for normalization. Because RNA was extracted from a pooled sample of 10 individual plants, the qRT-PCR results represent the average expression of the sampled population, with three technical replicates per tissue. All primer sequences used for qRT-PCR are provided in Table S2.

### 4.4. Expression Heatmap Analysis

The relative expression levels were calculated using the 2^−ΔΔCt^ method. The average expression of *OsMTB2* in stem was used as the calibrator, with its ΔΔCt value set to 0. For heatmap visualization, expression values of each gene were log_2_-transformed and plotted across root, stem, seed, callus, tiller bud, and panicle tissues. The heatmap was generated online in BioLadder (https://www.bioladder.cn/ (accessed on 28 July 2026)).

### 4.5. Plasmid Construction

Full-length CDS of *OsMTA*, *OsMTB1* and *OsMTB2* were used for all construct preparations.

For CRISPR/Cas9 knockout constructs, two sgRNAs were designed for each gene using the CRISPR-GE online tool (https://skl.scau.edu.cn/ (accessed on 28 July 2026)). The target sequences are indicated in the sequence alignment figures (Figure 4A and Figure S1). The sgRNAs were cloned into pYLCRISPR/Cas9 Pubi-H using Golden Gate cloning [29].

For RNAi constructs, each CDS was cloned into pZHY932 pre-digested with KpnI and SmaI using the In-Fusion kit (Clontech, Takara Bio, Mountain View, CA, USA).

For overexpression constructs, the full-length CDS of each gene was cloned into pRHVcGFP driven by the maize ubiquitin promoter. The vector was pre-digested with BamHI and SacI, and the fragments were inserted using the In-Fusion kit (Clontech).

For Y2H assays, bait and prey constructs were generated using pGBKT7 (BD) and pGADT7 (AD) vectors. The vectors were pre-digested with BamHI and EcoRI, and the CDS fragments were inserted using the In-Fusion kit (Clontech).

For BiFC assays, plant transformation constructs were prepared using pSAT vectors [30]. The restriction sites used for BiFC were XhoI and KpnI, with DNA fragments inserted using the In-Fusion kit (Clontech) following the manufacturer’s instructions.

For luciferase complementation imaging (LCI) assays, the CDS of each protein was inserted into pCAMBIA1300-split-nLUC and pCAMBIA1300-split-cLUC vectors pre-digested with BamHI and KpnI using the In-Fusion kit (Clontech).

### 4.6. Subcellular Localization

Seeds of overexpression lines were germinated at 28 °C for 5 days. Root tips (1 cm in length) were excised from germinated seeds, and a 1 mm section from the root tip was cut using a scalpel. The tissue was placed on a glass slide, covered with a coverslip, and gently pressed. Fluorescence was captured using a Nikon A1 confocal microscope with excitation at 488 nm (Tokyo, Japan).

### 4.7. Luciferase Complementation Imaging (LCI) Assay

Recombinant plasmids were transformed into *Agrobacterium tumefaciens* strain GV3101. *Nicotiana benthamiana* leaves (18–21 days old) were used for transient expression. *Agrobacterium* cultures were grown to OD_600_ = 0.4–0.6, harvested by centrifugation at 4700 rpm for 10 min, and resuspended in infiltration buffer (10 mM MES, 10 mM MgCl_2_, 150 μM acetosyringone, pH 5.6) to an OD_600_ of 0.5. Strains carrying different constructs were mixed in equal proportions and incubated at room temperature for 2–3 h before infiltration into N. benthamiana leaves using a needleless syringe. Infiltrated plants were grown for 2–3 days.

For luminescence detection, D-luciferin sodium salt was dissolved in deionized water to a working concentration of 1 mM. The solution was evenly applied to the abaxial side of the infiltrated leaf area. After incubation in the dark for 8 min, luminescence was captured using a ChampChemi 580 chemiluminescence imaging system (SageCreation, Beijing, China).

### 4.8. Bimolecular Fluorescence Complementation (BiFC) and Confocal Microscopy

To analyze the *in planta* interactions between OsMTA and OsMTB1/OsMTB2, agrobacterium strains harboring BiFC constructs (OsMTA-P2YC, OsMTB1-P2YN or OsMTB2-P2YN, and the empty controls P2YC and P2YN) were transiently co-transformed into *Nicotiana benthamiana* leaves by infiltration following established protocols [31]. After 2 days, YFP fluorescence was observed using a Nikon A1 confocal microscope with 488 nm laser excitation and 525 nm emission detection.

### 4.9. Yeast Two-Hybrid (Y2H) Assay

Y2H assays were performed using the Y2H Gold-Gal4 system (Clontech). BD and AD constructs were co-transformed into Y2H Gold yeast cells by the lithium acetate-mediated method. The transformed yeast strains were grown on SD/-Leu-Trp medium at 28 °C for 3 days. Protein interactions were assessed by growth on SD/-Leu-Trp-His-Ade medium at 28 °C for another 3 days.

### 4.10. mRNA Purification, Digestion, and m^6^A Quantification by LC-MS/MS

For m^6^A quantification, the same BiFC transient expression system described in Section 4.8 was used. Each infiltration treatment was performed with three independent biological replicates. Leaf samples were collected two days after infiltration. Total RNA was extracted as described in Section 4.3. RNA concentration and purity were assessed using a NanoDrop One spectrophotometer (Thermo Fisher Scientific). mRNA was purified using the Dynabeads mRNA DIRECT Kit (Thermo Fisher Scientific) following the manufacturer’s instructions. This poly(A)-based purification removes the vast majority of rRNA and genomic DNA. Purified mRNA (200 ng) was digested in a 40 µL reaction containing 0.4 µL Nucleoside Digestion Mix and 4 µL 10× Nucleoside Digestion buffer at 37 °C for 1 h, followed by addition of 60 µL DEPC-treated water. The digested products were then sent to the Testing Center of South China Agricultural University for m^6^A abundance analysis using UPLC-MS/MS.

UPLC-MS/MS analysis was performed using an ACQUITY UPLC BEH C18 column (2.1 mm × 50 mm, 1.7 μm; Waters, Milford, MA, USA) coupled to an ACQUITY TQD triple-quadrupole mass spectrometer (Waters, Milford, MA, USA). Mobile phase A was acetonitrile containing 0.01% formic acid, and mobile phase D was ultrapure water containing 0.01% formic acid. The gradient elution program was as follows: 0–2.40 min, 0.2% A; 2.40–3.80 min, 0.2–0.8% A; 3.80–5.20 min, 0.8–1.8% A; 5.20–6.60 min, 1.8–3.2% A; 6.60–7.00 min, 3.2–8.0% A; 7.00–9.00 min, 8.0–0.2% A; 9.00–15.00 min, 0.2% A. The flow rate was 0.100 mL/min, and the injection volume was 3 μL. Mass spectrometry was performed in positive ion mode. Nucleosides were quantified by mass transitions from nucleoside to base ions: *m*/*z* 268.0→136.0 for adenosine (A) and *m*/*z* 282.0→150.1 for m^6^A.

### 4.11. Phenotypic Analysis

Heading date was recorded as the number of days from sowing to when the main panicle emerged 1–2 cm from the leaf sheath. For each line, 16 individual plants were measured for heading date, plant height, and tiller number. Eight of these plants were further used to determine seed-setting rate and 1000-grain weight. The seed-setting rate was calculated as the percentage of filled grains. 1000-grain weight was measured from 1000 dried grains. Grains from five plants were used to measure grain length and grain width.

For representative photographs, plants were uprooted from the field after phenotypic measurement, washed, and placed in pots for imaging. These images were for illustration only and not used for data collection.

### 4.12. Statistical Analysis

Statistical analyses were performed using GraphPad Prism 9.5.0 (GraphPad Software, La Jolla, CA, USA). Phenotypic data were analyzed by one-way ANOVA followed by Dunnett’s test, with each transgenic line compared against the wild-type ZH11 control. The m^6^A/A ratio data were analyzed by one-way ANOVA followed by LSD test. qRT-PCR data and the phenotypic comparison of *osmtb2-ko2* versus ZH11 were analyzed by Student’s *t*-test. Data are presented as mean ± SD. Significance levels are indicated as * *p* < 0.05 and ** *p* < 0.01.

## Data Availability

The original contributions presented in this study are included in the article/Supplementary Materials. Further inquiries can be directed to the corresponding authors.

## Supplementary Materials

The following supporting information can be downloaded at: https://www.mdpi.com/article/10.3390/plants15172667/s1, Figure S1. Sequence alignment of *OsMTA* and additional *OsMTB2* transgenic lines. Figure S2. PCR detection of *Cas9* transgene in *OsMTB1* and *OsMTB2* knockout lines. Figure S3. Original Sanger sequencing results of knockout lines. Figure S4. Fluorescence observation of *OsMTB1* overexpression lines. Figure S5. Agronomic traits of *osmtb2-ko2*. Table S1. Heading date of *OsMTB1-OE1* versus ZH11 in crosses with HHZ (Huanghuazhan). Figure S6. Heading phenotype of *OsMTB1-OE1* versus ZH11 in crosses with HHZ. Table S2. The primers used for qRT-PCR.

## References

[1] Desrosiers R., Friderici K., Rottman F. (1974). Identification of Methylated Nucleosides in Messenger RNA from Novikoff Hepatoma Cells. Proc. Natl. Acad. Sci. USA.

[2] Sordyl D., Boileau E., Bernat A., Maiti S., Mukherjee S., Moafinejad S.N., Farsani M.A., Shavina A., Cappannini A., Agostini G. (2025). MODOMICS: A database of RNA modifications and related information. 2025 update and 20th anniversary. Nucleic Acids Res..

[3] Yang Y., Hsu P.J., Chen Y.S., Yang Y.G. (2018). Dynamic transcriptomic m^6^A decoration: Writers, erasers, readers and functions in RNA metabolism. Cell Res..

[4] Zhong S., Li H., Bodi Z., Button J., Vespa L., Herzog M., Fray R.G. (2008). MTA Is an *Arabidopsis* Messenger RNA Adenosine Methylase and Interacts with a Homolog of a Sex-Specific Splicing Factor. Plant Cell.

[5] Liu J., Yue Y., Han D., Wang X., Fu Y., Zhang L., Jia G., Yu M., Lu Z., Deng X. (2014). A METTL3–METTL14 complex mediates mammalian nuclear RNA N6-adenosine methylation. Nat. Chem. Biol..

[6] Wang G., Li H., Ye C., He K., Liu S., Jiang B., Ge R., Gao B., Wei J., Zhao Y. (2024). Quantitative profiling of m^6^A at single base resolution across the life cycle of rice and *Arabidopsis*. Nat. Commun..

[7] Khanzada H., Wassan G.M., Wang P., Khanzada S., Wang X., Xia Z. (2026). Comprehensive Genome-Wide Identification and Expression Analysis of the N^6^-Methyladenosine (m^6^A) Regulatory Network Influences Rapid Stress Adaptation with Exogenous Melatonin in Rice. J. Pineal Res..

[8] Zhang F., Zhang Y.C., Liao J.Y., Yu Y., Zhou Y.F., Feng Y.Z., Yang Y.W., Lei M.Q., Bai M., Wu H. (2019). The subunit of RNA N6-methyladenosine methyltransferase OsFIP regulates early degeneration of microspores in rice. PLoS Genet..

[9] Ma K., Han J., Zhang Z., Li H., Zhao Y., Zhu Q., Xie Y., Liu Y.G., Chen L. (2021). OsEDM2L mediates m^6^A of EAT1 transcript for proper alternative splicing and polyadenylation regulating rice tapetal degradation. J. Integr. Plant Biol..

[10] Tang J., Lei D., Yang J., Chen S., Wang X., Huang X., Zhang S., Cai Z., Zhu S., Wan J. (2024). OsALKBH9-mediated m^6^A demethylation regulates tapetal PCD and pollen exine accumulation in rice. Plant Biotechnol. J..

[11] Yang J., Chen Z., Wang P., Nyasulu M., Cheng Q., He X., Xu J., Fu J., Zhou D., Ouyang L. (2025). The critical role of OsYTH10 in promoting early flowering of rice under long sunlight. Theor. Appl. Genet..

[12] Cui S., Song P., Wang C., Chen S., Hao B., Xu Z., Cai L., Chen X., Zhu S., Gan X. (2024). The RNA binding protein EHD6 recruits the m^6^A reader YTH07 and sequesters OsCOL4 mRNA into phase-separated ribonucleoprotein condensates to promote rice flowering. Mol. Plant.

[13] Huang Y., Zheng P., Liu X., Chen H., Tu J. (2021). OseIF3h Regulates Plant Growth and Pollen Development at Translational Level Presumably through Interaction with OsMTA2. Plants.

[14] Yu Q., Liu S., Yu L., Xiao Y., Zhang S., Wang X., Xu Y., Yu H., Li Y., Yang J. (2021). RNA demethylation increases the yield and biomass of rice and potato plants in field trials. Nat. Biotechnol..

[15] Li Y., Yin M., Wang J., Zhao X., Xu J., Wang W., Fu B. (2025). Epitranscriptome profiles reveal participation of the RNA methyltransferase gene OsMTA1 in rice seed germination and salt stress response. BMC Plant Biol..

[16] Chen X., Zhang Y., Ye Z., Xiang J., He S., Fang Y., Zhang X., Xue D. (2025). Genome-wide identification of AlkB homologous gene family and its expression patterns in rice under drought stress. Plant Growth Regul..

[17] Hou Y., Kong X., Li J., Liu C., Wang S., Xie S., Wang J., Liu H., Lei L., Zheng H. (2025). The m^6^A Methylation Profile Identified That OsHMT9.1 Deregulates Chromium Toxicity in Rice (*Oryza sativa* L.) Through Negative Regulatory Functions. Agriculture.

[18] He S., Xiang J., Wang R., Ye Z., Zhang Y., Fang Y., Zhang X., Zhang J., Chen X., Xue D. (2026). Genome-wide identification of m^6^A methyltransferase genes and m^6^A modification participates in the response to cold stress in rice. Front. Plant Sci..

[19] Ma N., Song P., Liu Z., Li Y., Cai Z., Ding M., Ma X., Xu Q., Yue Y., Luo T. (2025). Regulation of m^6^A RNA reader protein OsECT3 activity by lysine acetylation in the cold stress response in rice. Nat. Plants.

[20] Qadir M., Qin Y., Wang H., Nabi F., Wu J. (2025). Integrative genomic and transcriptomic analysis reveals the regulatory role of m^6^A genes in heat stress adaptation across rice genotypes. Plant Physiol. Biochem..

[21] Zhang K., Zhuang X., Dong Z., Xu K., Chen X., Liu F., He Z. (2021). The dynamics of N6-methyladenine RNA modification in interactions between rice and plant viruses. Genome Biol..

[22] Li S., Tan X.Y., He Z., Shen C., Li Y.L., Qin L., Zhao C.Q., Luo G.H., Fang J.C., Ji R. (2024). The dynamics of N6-methyladenine RNA modification in resistant and susceptible rice varieties responding to rice stem borer damage. Insect Sci..

[23] Hasan M., Nishat Z.S., Hasan M.S., Hossain T., Ghosh A. (2024). Identification of m^6^A RNA methylation genes in *Oryza sativa* and expression profiling in response to different developmental and environmental stimuli. Biochem. Biophys. Rep..

[24] Růžička K., Zhang M., Campilho A., Bodi Z., Kashif M., Saleh M., Eeckhout D., El-Showk S., Li H., Zhong S. (2017). Identification of factors required for m^6^A mRNA methylation in *Arabidopsis* reveals a role for the conserved E3 ubiquitin ligase HAKAI. New Phytol..

[25] Shen L., Liang Z., Gu X., Chen Y., Teo Z.W.N., Hou X., Cai W.M., Dedon P.C., Liu L., Yu H. (2016). N6-Methyladenosine RNA Modification Regulates Shoot Stem Cell Fate in *Arabidopsis*. Dev. Cell.

[26] Yang L., Wang B., Zhao D., Li X., Qin Y., Ouyang N., Xiao Z., Zhang Z., Galili G., Li J. (2024). Selective recognition of PTRE1 transcripts mediated by protein–protein interaction between the m^6^A reader ECT2 and PTRE1. Plant Commun..

[27] Kim J., Shim S., Lee H., Seo P.J. (2020). m^6^A mRNA Modification as a New Layer of Gene Regulation in Plants. J. Plant Biol..

[28] Sommer S., Lavi U., Darnell J.E. (1978). The absolute frequency of labeled N-6-methyladenosine in HeLa cell messenger RNA decreases with label time. J. Mol. Biol..

[29] Ma X., Liu Y. (2016). CRISPR/Cas9-Based Multiplex Genome Editing in Monocot and Dicot Plants. Curr. Protoc. Mol. Biol..

[30] Tzfira T., Tian G.W., Lacroix B., Vyas S., Li J., Leitner-Dagan Y., Krichevsky A., Taylor T., Vainstein A., Citovsky V. (2005). pSAT vectors: A modular series of plasmids for autofluorescent protein tagging and expression of multiple genes in plants. Plant Mol. Biol..

[31] Sparkes I.A., Runions J., Kearns A., Hawes C. (2006). Rapid, transient expression of fluorescent fusion proteins in tobacco plants and generation of stably transformed plants. Nat. Protoc..

