# Supplementary material for: Identification of Rice m6A Methyltransferase Complex Subunits MTA/MTB and Their Effects on Heading Date and Grain Weight
Source: Plants (Basel). 2026 Aug 31;15(17):2667. doi: 10.3390/plants15172667 (PMC13567530; doi:10.3390/plants15172667)
Supplement: Supplementary file 1 [file plants-15-02667-s001.zip › plants-4475198-supplementary.pdf]

Supplementary Materials for

Identification of Rice m^{6}A Methyltransferase Complex Subunits MTA/MTB and Their Potential Roles in Heading Date and Grain Weight

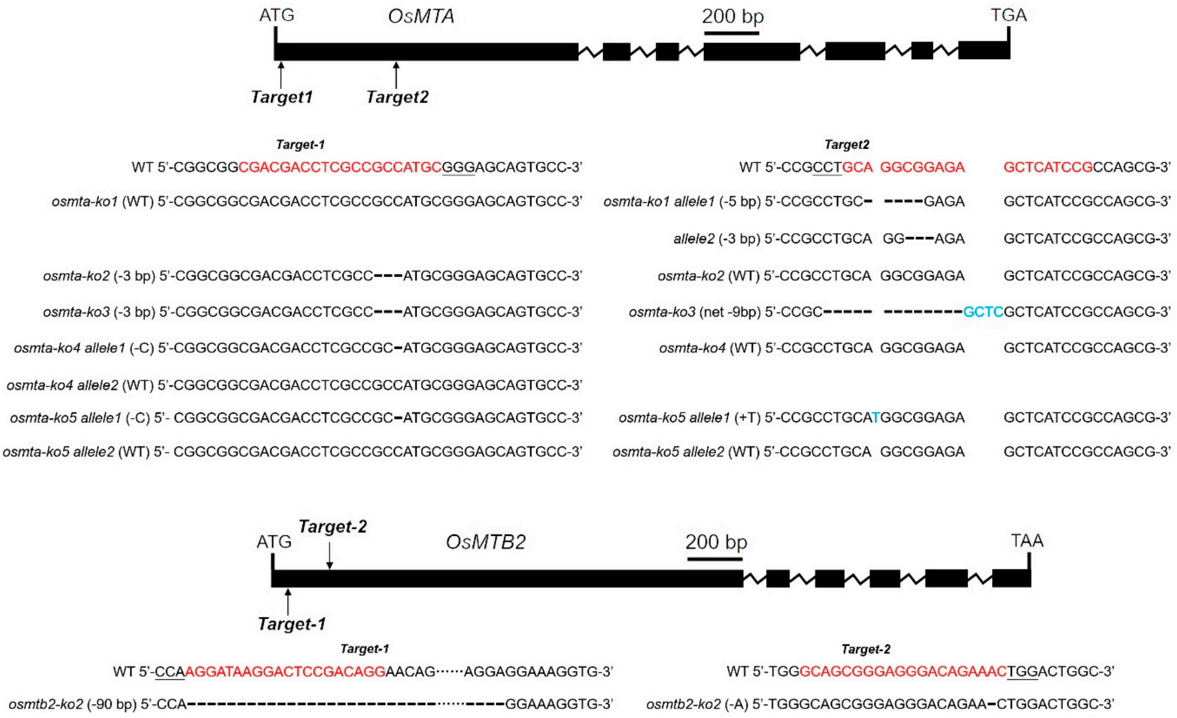

Figure S1. Sequence alignment chromatograms of of *OsMTA* and additional *OsMTB2* transgenic lines. The sequencing results revealed a 90-bp deletion accompanied by an adenine deletion in *osmtb2-ko2*. No homozygous frameshift mutations were observed in the *OsMTA* knockout lines. The target sites are highlighted in red. Heterozygous sequences were manually decoded from the Sanger chromatograms (Figure S2).

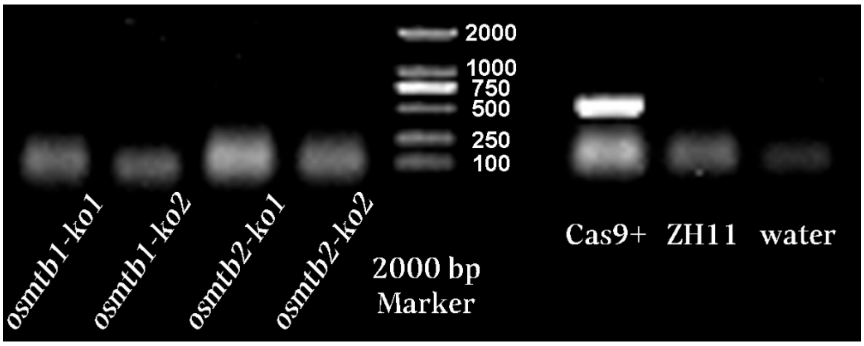

Figure S2. PCR detection of *Cas9* transgene in *OsMTB1* and *OsMTB2* knockout lines. The absence of *Cas9*-specific band indicates that the *Cas9* construct was removed.

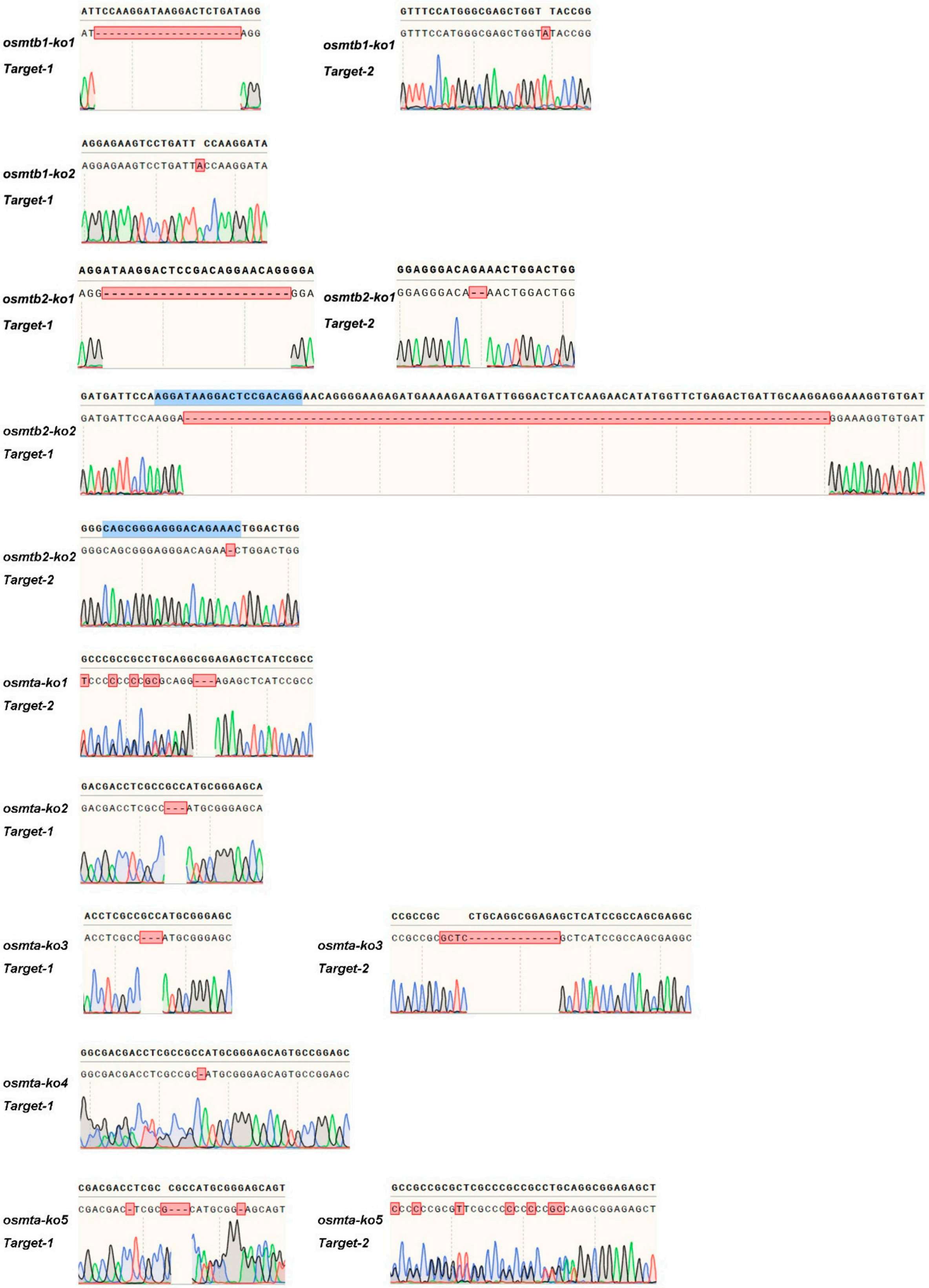

Figure S3. Original Sanger sequencing results of knockout lines.

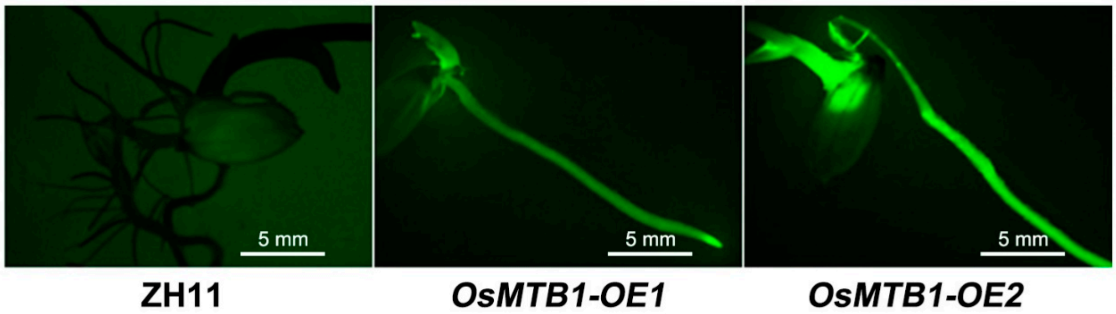

Figure S4. Fluorescence observation of *OsMTB1* overexpression lines.

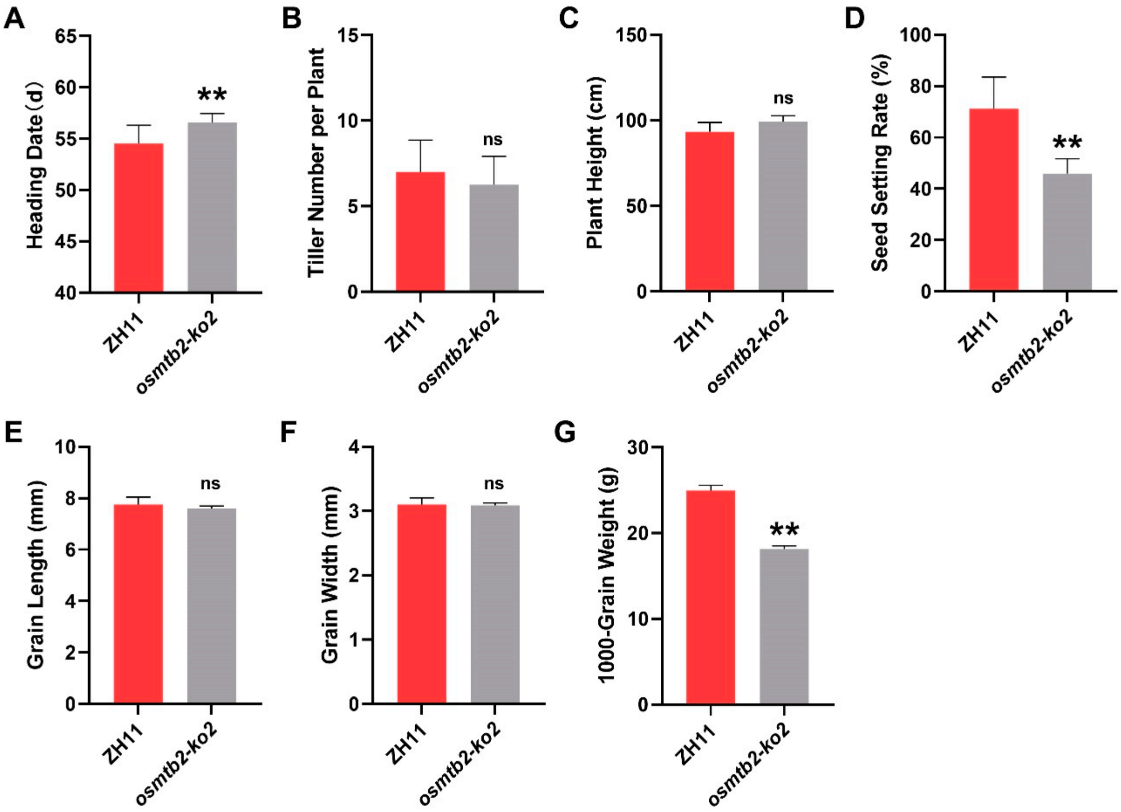

Figure S5. Agronomic traits of *osmtb2-ko2*. (**A**) Heading date, (**B**) Tiller number per plant, (**C**) plant height, (**D**) seed setting rate, (**E**) grain length, (**F**) grain width, (**G**) 1000-grain weight of *osmtb2-ko2* compared with ZH11. Data were collected in a separate growth season (2026, March to July; average daily temperature 19–32°C), presented as mean ±SD. Significant differences were determined by Student's t-test, **p<0.01, ns = no significance.

Table S1. Heading date of *OsMTB1-OE1* versus ZH11 in crosses with HHZ (Huanghuazhan)

Material	Heading date (d)
ZH11♀×HHZ♂	66.5±1.7
OsMTB1-OE1♀×HHZ♂	64.0±0.8**

Data are means ±SD. Significant differences were determined by Student's t-test, $p<0.01$

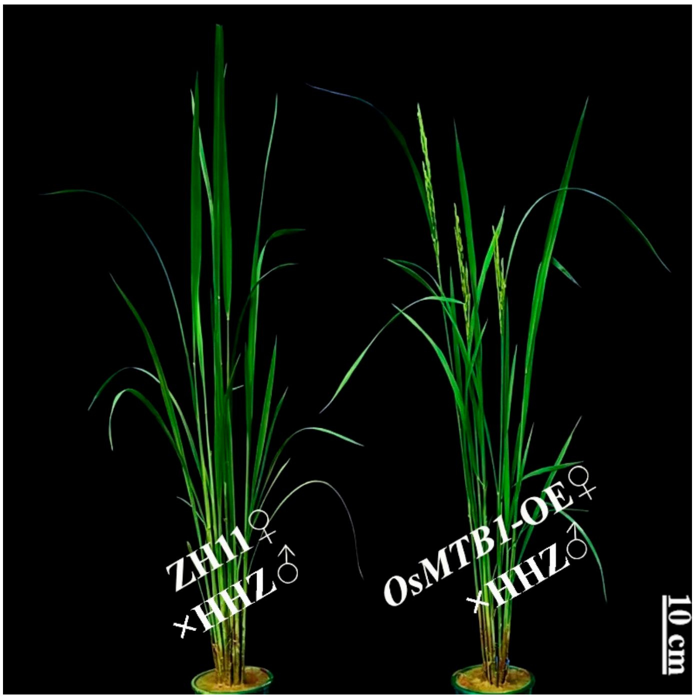

Figure S6. Heading phenotype of *OsMTB1-OE1* versus ZH11 in crosses with HHZ.

Table S2. The primers used for qRT-PCR

Gene name	Primer name	Primer sequence (5' to 3')
OsMTA	*OsMTA-q-F*	AAGTCCGTGAAACAAGCA
	OsMTA-q-R	TACCCAGAGAAAGCCATC
OsMTB1	*OsMTB1-q-F*	CTCTTTGGAGAGGATCATAA
	OsMTB1-q-R	TTTCCCATCCTTGTCTGCAA
OsMTB2	*OsMTB2-q-F*	TAGCCGATGAACCAACCG
	OsMTB2-q-R	CCCAAGAGTAAGCCAACCC
OsActin1	*OsActin-F*	GCTATGTACGTCGCCATCCAG
	OsActin-R	AATGAGTAACCACGCTCCGTCA
